# Life in the Indian Medical Service: The Attractions of Medicine and Sport

**Published:** 1914-09-05

**Authors:** 


### LIFE IN THE INDIAN MEDICAL SERVICE.

### The Attractions of Medicine and Sport.

The Indian Medical Service offers an interesting and varied career to any young medical man who does not dislike the prospect of nominal exile. The facilities for returning to England are so great, the time of the journey so shortened, that it is possible to spend a month in London out of two months' leave. The days in which fabulous fortunes could be acquired in the Service have passed, but still there are plums for the picking. The civil surgeons of the larger stations make, from official emoluments and the earnings of private practice, incomes that would make Harley Street envious were the amount divulged, and there are teaching billets in the Indian medical schools that compare in opportunity with similar posts in the big medical schools of Britain. It is not everyone that can win the big prizes, but, given health, there is a living for all who accept the Service, and the number of possible careers is large.

Every man commences by being attached for a time to a military hospital, and usually to a Royal Army Medical Corps mess, that he may learn something of Army ways and Indian methods before he is launched. But it is seldom many weeks before he is required for the temporary charge of a regiment. Off he goes to meet, usually, a kindly and hospitable welcome from his new brother officers and to do a little light work in the wards of a small regimental hospital that will strike him as totally inadequate to the treatment of the sick. Yet in it he will treat malaria and guinea-worm with some success and get results from operations that will often surprise him. Dry air, bright sunshine, and sparsely fed bodies are efficient allies to imperfect antiseptic measures. The young doctor early in his career probably will put down his name for civil employ. It is, however, open to him to elect to remain a regimental surgeon for all his service. This course he will not choose unless he is unambitious and satisfied with a lesser pay. To the man suited to it the life is, however, often very agreeable. There is little work and large leisure for the pursuit of sport and hobbies. It is a life though in which it is very possible to rust.

In civil employ a man may find himself at anything from his earliest days. He may be fighting plague; he may be in charge of a small station in the jungles with scarcely a white man to speak to; he may be the ruler of a gaol and become an adept at judicial hangings; he may be in a gay and busy military station looking after the civil population. But, wherever he may be, he will be in a country full of interest, with often good opportunities for sport, and with always unlimited scope for research into the origin and nature of disease, for operative work amongst a population that presents an amount of clinical material that cannot be conceived in this country, and for the researches of a naturalist amongst a fauna and flora of infinite and beautiful variety, if his tastes lie that way. His income will steadily progress with his rank and years of service, but different appointments bring different emoluments and the opportunities of making income by private practice vary with the station and the manner of man. He may, however, reckon that £400 a year is the least he will ever draw, and that he easily may be making twice that sum after a few years' service.

But there are also drawbacks to the Indian Medical Service that may be illusions, but that appear very real. The climate of India is not ideal in any part. It is not the same in every part, a fact not often realised by those without real Indian experience. The writer of this article cannot speak for the south; he has had long experience only of that district known as the United Provinces of Agra and Oudh and of the hill-stations north of it. He found the latter part of the hot weather hard to bear and the latter half of the rains perhaps worse. But the cold weather is delightful. For six months of the year India is a delightful country to live in. A jolly climate, plenty of work, plenty of games, and a good deal of sport, when a man has leisure to seek it. There are few joys in life to equal a good stand, with a good gun, amongst the high reeds in an Indian jheel (jheel = a marsh), the sun setting in a sky all red and gold and the duck flighting in over your head in hundreds. Life is worth living then. There are few excitements more intense than the dash through the high grass and jhow (jhow = a scrubby kind of tamarisk) of the Ganges Kardah with a bold horse between your thighs and a great grey boar slipping along in front of you.

But for six months the climate is detestable. Days and nights with the temperature round about 100° F. in the shade, with the punkahs constantly flapping, the mosquitoes ceaselessly humming, the prickly heat irritating your skin, and sand storms suddenly roaring up and upsetting all your arrangements. Hot steamy days when there comes a break in the rains and miserable periods when all your servants are down with fever. Life seems not worth living then. Yet somehow the days are got through and not altogether unpleasantly. It is much a matter of health. Temperance and exercise, and temperance in exercise during the hot weather. That is the secret. Health means so much in India. If a man of the Indian Medical Service cannot stand India he is done. His work is in India and nowhere else. Many of the stations are dull and dreary to a man not exceptionally full of resource, and all but the exceptionally lucky must do time, a fitting expression, at some period of their career in dull and unhealthy places. Yet few men leave it without a regret.

Note.

The Regulations for admission to the Indian Medical Service, with specimen examination papers and full details of the pay and appointments, can be obtained free on application to the Military Secretary, the India Office, Whitehall.

